# Plastic but not progressive changes in cognitive function and hippocampal volume in an adolescent with bipolar disorder: a case report

**DOI:** 10.3389/fpsyt.2024.1507333

**Published:** 2025-01-07

**Authors:** Bo Liu, Hui Sun, Qiannan Zhao, Li Li, Rong Tian, Su Lui, Hongru Zhu

**Affiliations:** ^1^ Mental Health Center, West China Hospital, Sichuan University, Chengdu, China; ^2^ Department of Psychosomatic Medicine, Zigong Mental Health Center, Zigong, China; ^3^ Department of Radiology, and Functional and Molecular Imaging Key Laboratory of Sichuan Province, West China Hospital of Sichuan University, Chengdu, China; ^4^ Huaxi MR Research Center (HMRRC), West China Hospital of Sichuan University, Chengdu, China; ^5^ Research Unit of Psychoradiology, Chinese Academy of Medical Sciences, Chengdu, China; ^6^ Department of Nuclear Medicine, West China Hospital, Sichuan University, Chengdu, China

**Keywords:** bipolar disorder, cognitive function, hippocampal volume, adolescent, case report, plasticity

## Abstract

Bipolar disorder (BD) is a prevalent mood disorder characterized by alternating episodes of depression and mania, often accompanied by varying degrees of cognitive impairment. Cognitive impairments often serve as indicators of a bleak prognosis or the likelihood of progressing to dementia. Additionally, some studies suggest that individuals diagnosed with BD may undergo a decline in hippocampal volume. However, the potential for reversibility of these changes, particularly in adolescents, remains unclear. We present an intriguing case involving an 18-year-old male student who experiences concurrent occurrences of both BD and mild cognitive impairment (MCI), accompanied by a subtle reduction in hippocampal volume. Initially, the individual exhibited impaired general cognitive function, as indicated by an IQ score of 80 on the Standard Raven’s Progressive Matrices test, and demonstrated slightly reduced bilateral hippocampal volume compared to the normative reference, as determined through quantitative structural magnetic resonance imaging (qsMRI). The deposition profiles of amyloid beta (Aβ) peptide in the brain were not identified with 18F-AV45 PET/MRI. Following six months of combined psychopharmacological treatment and cognitive behavioral therapy, the individual’s psychopathological symptoms improved significantly, leading to a restoration of his IQ score to 116 and normalization of hippocampal volume. This case suggests that the hippocampal volume reduction and cognitive impairment seen in some adolescents with BD may demonstrate greater plasticity compared to neurodegenerative conditions such as Alzheimer’s disease (AD). These findings highlight the potential importance of early intervention in young BD patients with cognitive impairments.

## Introduction

Bipolar disorder (BD) is characterized by recurrent mood disturbance, oscillating between depressive and manic episodes (1). Recent data suggested that BD affects approximately 8 million individuals in the United States and an estimated 40 million people worldwide (1). A recent meta-analysis reported that the estimated point prevalence and lifetime prevalence of BD in China were 0.09% and 0.11%, respectively (2). The clinical presentations of BD can exhibit notable variability both among individuals and within the lifespan of affected individuals (3). BD encompasses a group of highly heterogeneous mental illnesses primarily characterized by mood symptoms, sometimes accompanied by psychotic symptoms, and varying levels of cognitive impairment. In recent years, there has been increased attention paid to the cognitive impairment associated with BD, as it tends to predict poorer outcomes or progression to dementia (4). Despite not being the most prominent clinical symptoms, cognitive impairments in BD are often overlooked by psychiatrists in clinical practice unless they are clearly apparent. Patients diagnosed with BD often experience cognitive and functional impairments before, during, and after the onset of the disease, which can significantly impact their overall quality of life (5–7). A recent systematic review has shown that individuals with BD, even during stable phases, demonstrate cognitive impairments across various domains, such as attention, conceptual thinking, motor skills, executive functioning, and visuospatial abilities (8). While there is evidence suggesting that the severity of cognitive impairments in individuals with BD may serve as a predictor of a less favorable prognosis (9), the nature of cognitive impairment in BD remains a subject of debate, indicating cognitive heterogeneity within the disorder.

Mild cognitive impairment (MCI),also known as mild neurocognitive disorder, is defined by the impairment of at least one cognitive domain and represents an intermediate stage of cognitive decline between typical age-related cognitive changes and the onset of dementia (10). Impairments in attention and executive function are more common in BD, while cognitive impairments in MCI are more likely to involve memory and learning problems (8). Furthermore, the coexistence of both has been reported. However, the comorbidity of the two diseases is relatively rare in adolescents A previous study suggested that MCI in adults with BD may serve as a predictor for the subsequent development of dementia (4). Additionally, the hippocampus plays a crucial role in cognitive processing and hippocampal atrophy has been linked to both MCI and Alzheimer’s disease(AD) (11). While the evidence on hippocampal volume in BD is inconsistent, a recent systematic review suggested a slight reduction in hippocampal volume in individuals with BD, particularly in cases of early-onset BD (12). Furthermore, evidence from a recent longitudinal work suggests that the alterations in hippocampal volumes in BD might be adaptive changes rather than indicative of progressive degeneration (13). However, the precise relationships between BD, MCI, and hippocampal atrophy are still a subject of ongoing research and debate.

In this context, we present an intriguing case of a male adolescent experiencing concurrent BD and MCI, accompanied by subtle hippocampal atrophy, yet without the deposition of amyloid beta (Aβ) peptide in cortical regions. Notably, we observed a simultaneous improvement in cognitive function, hippocampal volume, and clinical symptoms after six months of treatment.

## Case presentation

A 18-year-old student was admitted with a two-year history of emotional instability and 10 days of feelings of criticism. Throughout high school, he experienced jealousy and feelings of inferiority due to constant comparison of grades with peers. His psychiatric history began with disappointment over his performance on a high school admission examination. He manifested physical symptoms such as indigestion, bloating, constipation, and insomnia, along with psychological symptoms, including persistent low mood.

**Figure 1:**
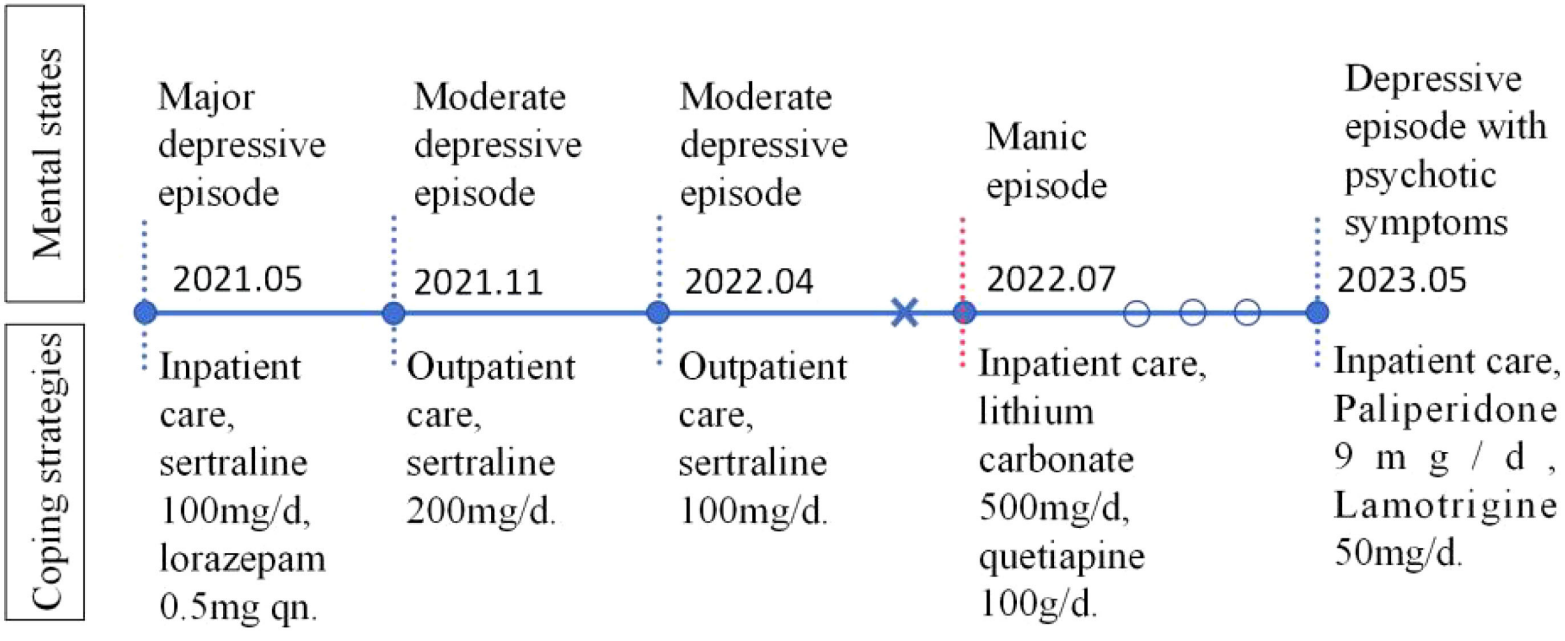
The timeline of the patient’s disease progression and medication history. The cross indicates the timepoint when the patient discontinued the medication, two months prior to the manic episode. The hollow circle indicates the patient took the medication intermittently.

The patient was initially diagnosed with a depressive episode and prescribed 50 mg of sertraline twice daily for treatment. After a month of medication, his depression significantly improved, enabling his return to school. Despite consistent medication in the first year, he experienced additional two episodes of moderate depression, necessitating a maximum sertraline dosage of 200 mg per day. After his depressive symptoms subsided, he stopped the medication for two months. However, he then suddenly developed manic symptoms, including an elevated mood, increased energy, excessive activity, and a tendency to initiate conversations. His score on the Young Mania Rating Scale was 20. Subsequently, he received a new diagnosis of BD and was prescribed 500 mg of lithium carbonate and 100 mg of quetiapine daily. This treatment helped stabilize his mood, enabling him to continue his education. However, he took the medication intermittently. Ten days prior to his current hospital admission, he began to perceive others discussing or talking about him. He also reported frequent episodes of zoning out, low energy levels, diminished interest in activities, and an inability to experience joy. Furthermore, he exhibited slow reactions, impaired concentration, and memory difficulties. These symptoms prompted his admission to the hospital. The detailed timeline of the disease course is presented in **Figure 1**.

Regarding his background, the subject is the only child in his family and had a typical upbringing with normal growth and development. He excelled academically, demonstrated a gentle temperament, was meticulous in his work, maintained harmonious relationships with his family, and did not experience any significant traumatic events. His father is a doctor, while his mother is currently unemployed. Although his mother previously struggled with postpartum depression, she has since recovered.

Upon admission, the physical examination did not reveal any abnormalities, while the mental examination indicated symptoms of depression, including persistent low mood and inability to experience happiness, suggestive of depressive syndrome. Psychotic symptoms such as feeling observed or persecuted were also present, along with attention and memory impairments. His complaints of poor memory and slow reactions prompted us to assess his cognitive function. The Perceived Deficits Questionnaire for Depression5 (PDQ-D5), initially intended to gauge perceived cognitive challenges in individuals with depression, and now also employed in assessing concentration, memory, decision-making, and attention among those with BD (14). The initial score on the PDQ-D5 was 16. The Standard Raven’s Progressive Matrices test showed an IQ of 80, indicating lower average intelligence.

**Figure 2:**
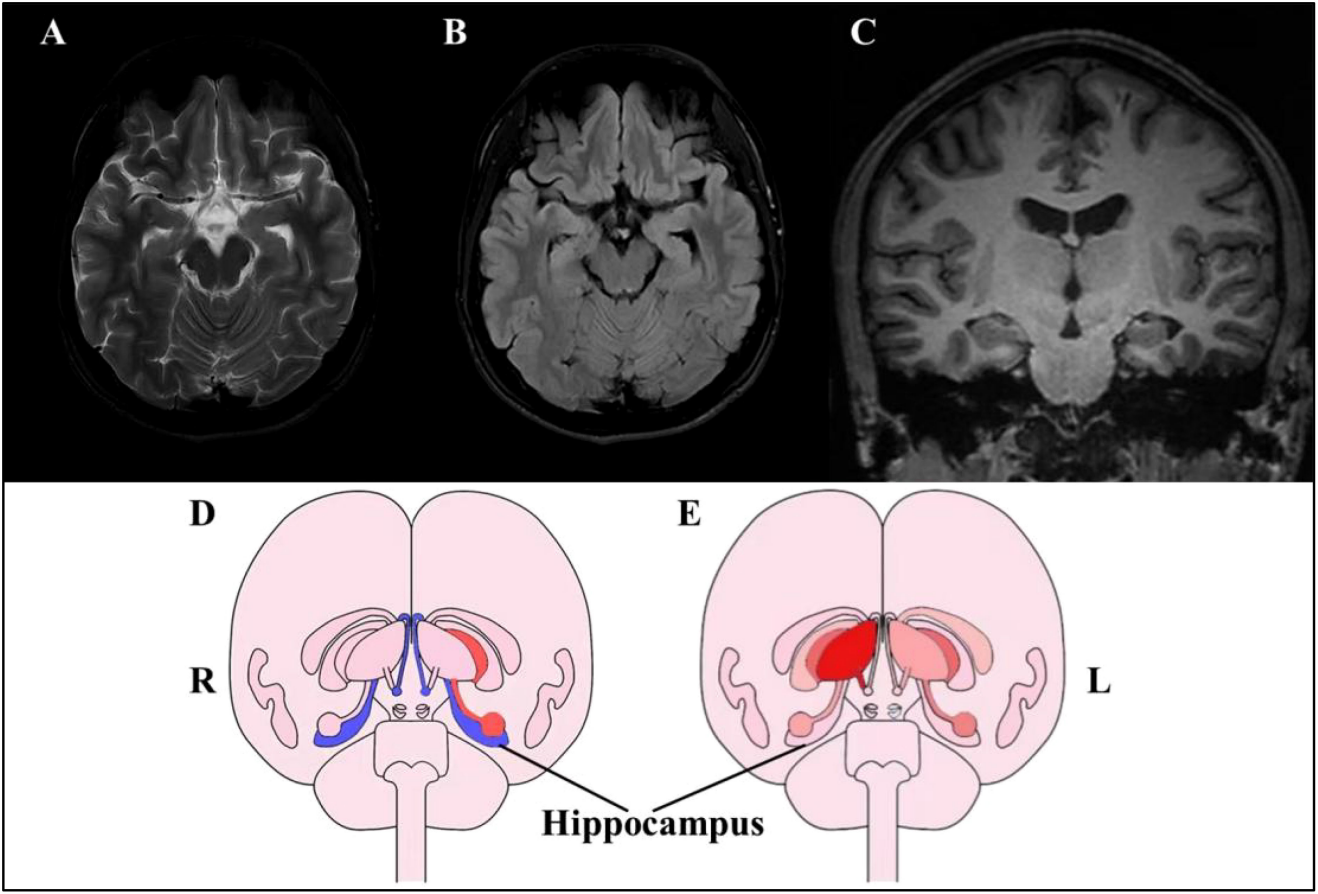
The MRI images of the patients. **(A-C)** conventional structural MRI, including T2-weighted **(A)**, T2-weighted and fluid-attenuated inversion recovery imaging **(B)**, and T1-weighted **(C)**, revealed no visible abnormalities. **(D, E)** indicate quantitative structural magnetic resonance imaging (qsMRI). The illustrations in **(D)** (the first scan, May 25,2023) and **(E)** (the second scan, Dec 19,2023) represent changes in the subcortical structures relative to the normative reference. The blue color indicates that the volume of the corresponding brain region was below the normative modeling charts for the corresponding age and sex, whereas the red color indicates a higher volume. A relatively lower volume of bilateral hippocampi was observed in **(D)** and recovery to normal level in **(E)**.

**Figure 3:**
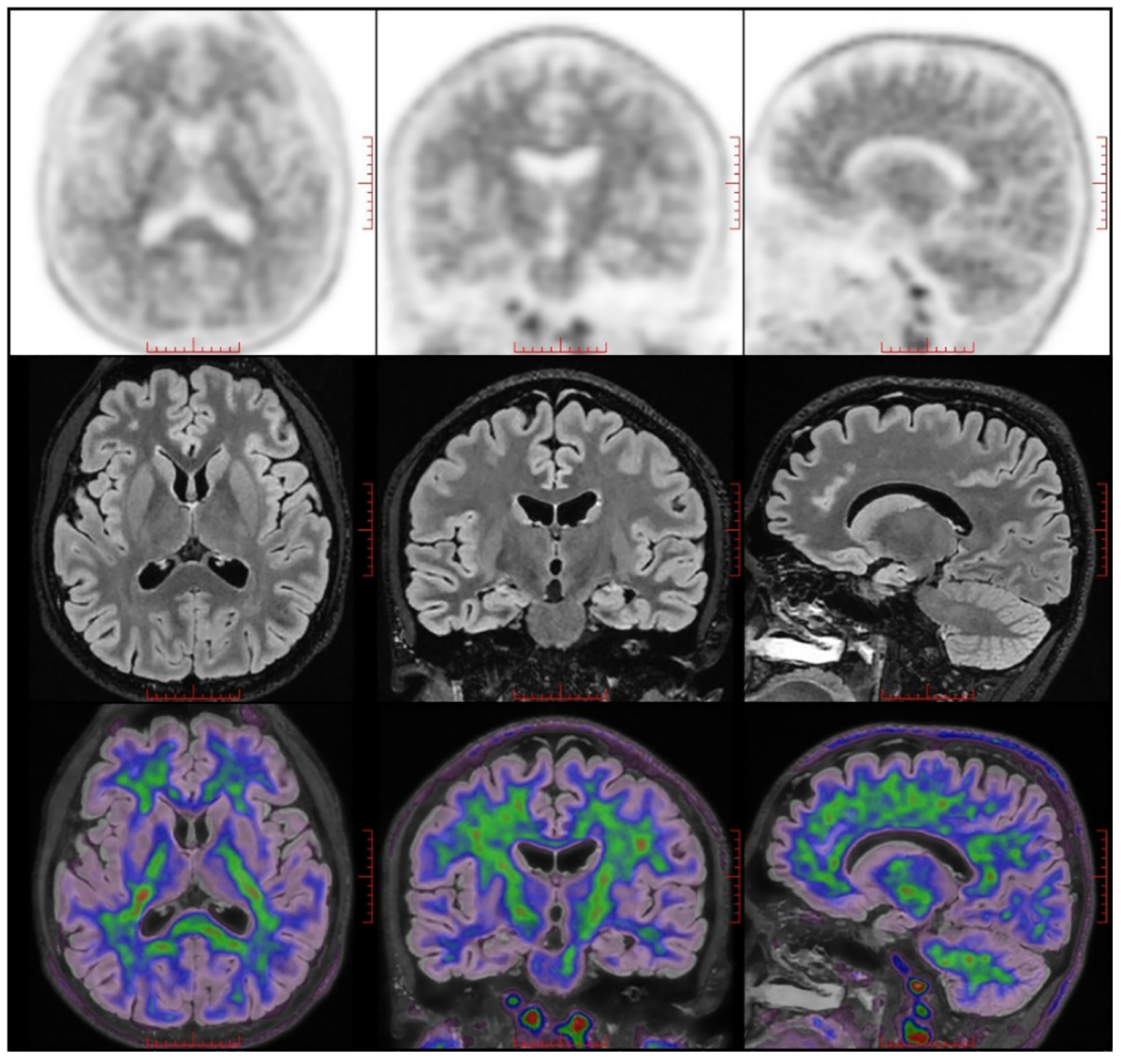
The PET/MRI images of the patient. ^18^F-AV45 PET/MRI showed no significant cortical Aβ deposition.

To investigate potential neurological factors contributing to the early-onset cognitive impairments, neuroimaging was conducted on the patient’s brain. Conventional magnetic resonance imaging (MRI) revealed no intracranial abnormalities, while quantitative structural MRI (qsMRI) showed bilateral reductions in hippocampal volume (**Figure 2**). A PET/MRI scan was performed 50 minutes after the intravenous injection of 18F-AV45 at 9.91 mCi to assess for amyloid-beta (Aβ) deposition. The results indicated no abnormal cortical Aβ deposition in the brain (**Figure 3**).

Based on the findings, the subject was diagnosed with concurrent bipolar disorder (BD) and mild cognitive impairment (MCI). He was prescribed a maximum daily dose of 9 mg of Paliperidone extended-release tablets and a maximum daily dose of 50 mg of Lamotrigine orally disintegrating tablets. Cognitive behavioral therapy sessions were conducted twice weekly during hospitalization. Following a 16-day hospitalization, the subject was discharged with significant clinical improvement in symptoms.

**Figure 4:**
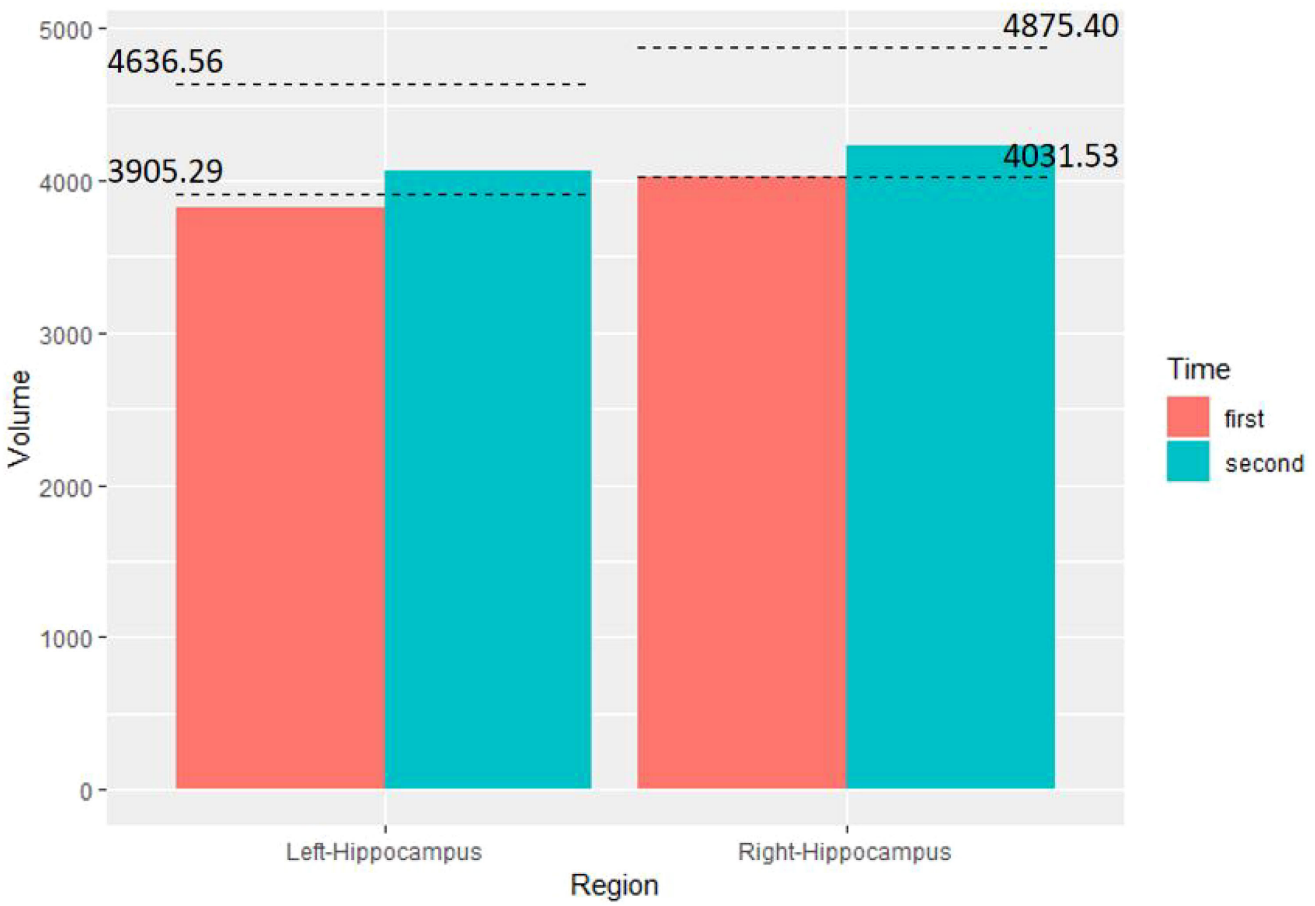
The changes in the hippocampal volume from qsMRI of the patient. The bar chart demonstrates the volume (mm3) of the bilateral hippocampus at two time point. The four dashed lines represent the normative lower and upper limits of the left hippocampus and right hippocampus for the corresponding age and sex, respectively.

Six months later, following a reduction in his psychopathological symptoms, the individual returned to school and successfully adapted to the academic environment. Subsequent reassessment of his cognitive abilities, including a repeat quantitative susceptibility magnetic resonance imaging (qsMRI) scan of his brain, revealed an increase in his IQ score on the Standard Raven’s Progressive Matrices test to 116 and a decrease in his PDQ-D5 score to 6. Furthermore, his bilateral hippocampal volume showed an increase compared to baseline, now falling within the normal range (**Figure 4**).

## Discussion

In the present case, we observed plastic, rather than progressive, changes in cognitive function and hippocampal volume in an adolescent with bipolar disorder. While cognitive impairments are commonly reported in adults with BD, a similar pattern of cognitive deficits has been noted in children and adolescents with the disorder (15). We observed a significant cognitive decline in the patient, particularly noteworthy given his previous academic performance. The patient’s PDQ-D5 and IQ scores confirmed the presence of cognitive impairments. Previous research has indicated a higher risk of dementia in older adults with a history of BD (16). Additionally, assessing cognitive impairment can help predict the onset of dementia in adults with BD (4). Therefore, we investigated whether the patient exhibited altered profiles in biomarkers associated with Alzheimer’s disease (AD).”Aβ deposition is a well-established biomarker of AD. However, our study did not detect any Aβ deposition in cortical regions, consistent with prior research showing that individuals with BD and cognitive impairments did not exhibit typical AD-related biomarkers. Specifically, lower levels of Aβ1-42 were observed, along with increased concentrations of total tau (T-tau) and tau phosphorylated at threonine 181 (P-tau) in the cerebrospinal fluid.” (17).

Regarding hippocampal volume, Bearden CE et al. found that adolescents with BD exhibited a significant 9.2% reduction in total hippocampal volume compared to controls (18). Our subject showed deficits in bilateral hippocampal volume at baseline, suggesting potential structural damage. The hippocampus is known to play a crucial role in various cognitive processes (19), and deficits in this area have been inconsistently reported in BD (12). This variability may indicate different subtypes within BD, with one subtype possibly associated with hippocampal damage. High-resolution structural MRI and quantitative analytical methods could aid in early identification of such subtypes.

Notably, the cognitive impairments and hippocampal atrophy observed in the subject improved to normal levels after six months of treatment. These plastic changes may be attributed to the treatment, as previous research suggests that certain mood stabilizers can impact hippocampal volume. For instance, Lithium has been linked to increased hippocampal volume in bipolar disorder, likely due to its neurotrophic effects (20). Similarly, lamotrigine, as an alternative mood stabilizer, may also have a similar effect on hippocampal volume (21), though further research is needed to confirm this hypothesis.

Our findings support the existence of multiple forms of Mild Cognitive Impairment (MCI), each potentially associated with distinct neuropathological correlates or underlying brain changes (22). In contrast to the progressive cognitive decline seen in AD, our case suggest that cognitive impairments and hippocampal atrophy observed through quantitative structural MRI in the subject may demonstrate greater plasticity (13). The various definitions and assessment methods for MCI may contribute to diagnostic heterogeneity (4). Therefore, a more accurate classification of MCI based on psychopathological symptoms and biomarkers from other dimensions, such as neuroimaging and molecular biological profiles, is needed.

From a psychosocial perspective, the patient exhibits both protective and risk factors. On the positive side, he comes from a warm family with a strong relationship with his parents. He has also demonstrated good academic performance since childhood and has no harmful habits. In contrast, his mother has a history of postpartum depression, and his parents tend to over-pamper him, which has contributed to his over-dependence on them. As a result, long-term psychotherapy is necessary to support his recovery.

## Limitation

Although we observed an interesting phenomenon — that changes in cognitive function and hippocampal volume in an adolescent with BD are plastic rather than progressive — this is just one case and cannot represent all adolescent BD patients. Interestingly, the patient experienced more depressive episodes than manic episodes, along with some psychotic symptoms, and his mother had postpartum depression, suggesting that this case may represent a subtype of mood disorders. To validate our hypothesis, long-term follow-up is needed.

## Conclusion

We present an interesting case of an adolescent with bipolar disorder (BD) demonstrating concurrent changes in clinical symptoms, cognitive function, and hippocampal volume. The underlying mechanisms remain unclear, but this case may represent a subset of BD with a favorable prognosis. This underscores the importance of utilizing structural and molecular imaging technologies to assess cognitive impairment in clinical settings. Long-term follow-up is essential to monitor progression and validate our hypothesis. A large cohort study of adolescent BD is needed to provide stronger evidence.

## Data Availability

The original contributions presented in the study are included in the article/supplementary material. Further inquiries can be directed to the corresponding author.
